# Planar Near-Field Measurements of Low-Sidelobe Antennas

**DOI:** 10.6028/jres.099.013

**Published:** 1994

**Authors:** Michael H. Francis, Allen C. Newell, Kenneth R. Grimm, John Hoffman, Helmut E. Schrank

**Affiliations:** National Institute of Standards and Technology, Gaithersburg, MD 20899-0001; Nichols Research Corporation, Vienna, VA 22182-2222; System Engineering Corporation, Columbia, MD 21046; Hunt Valley, MD 21030

**Keywords:** antenna measurements, far field, low-sidelobe antennas, planar near field

## Abstract

The planar near-field measurement technique is a proven technology for measuring ordinary antennas operating in the microwave region. The development of very low-sidelobe antennas raises the question whether this technique can be used to accurately measure these antennas. We show that data taken with an open-end waveguide probe and processed with the planar near-field methodology, including probe correction, can be used to accurately measure the sidelobes of very low-sidelobe antennas to levels of −55 dB to −60 dB relative to the main beam peak. A special probe with a null in the direction of the main beam was also used for some of these measurements. This special probe reduced some of the measurement uncertainties but increased the uncertainties due to probe-antenna interactions. We discuss the major sources of uncertainty and show that the probe-antenna interaction is one of the limiting factors in making accurate measurements. The test antenna for this study was a slotted-waveguide array whose low sidelobes were known. The near-field measurements were conducted on the NIST planar near-field facility.

## 1. Introduction

This paper describes the efforts of several groups of scientists and engineers to determine the sidelobes of a very low-sidelobe array antenna using the near-field technique and to quantify the uncertainties. More than 20 years ago the National Institute of Standards and Technology (then the National Bureau of Standards) [1–3] and other laboratories [4] developed the theory and basic measurement methods for using the planar near-field (PNF) technique to determine the far field of antennas. Newell and Crawford [5] compared the far fields of array antennas obtained using near-and far-field measurement techniques. This comparison indicated that there was good agreement between the directly measured far field and the far field as determined using the near-field technique. Nevertheless, little has been reported on the effectiveness of using the near-field technique to determine the far-field patterns of a very low-sidelobe antenna.

In Sec. 2 we describe the experiment, including a description of the array, the design of a special probe, and a description of the near-field measurement facility. Section 3 describes the diagnostic tests and Sec. 4 describes the final measurement results. In Sec. 5 we evaluate the uncertainties. In Sec. 6 we discuss possible future research. Finally, in Sec. 7 a summary is given.

## 2. Design of Experiment

### 2.1 Purpose of Experiment

This experiment had three objectives. The first was to determine whether the PNF technique could be used to accurately measure low-sidelobe arrays. In order to determine this, two low-sidelobe arrays, the Ultra-Low-Sidelobe Array (ULSA) and the Airborne Warning and Control System Array (AWACS), were measured using the PNF technique.

Second, we wanted to show that the PNF analyses of Yaghjian [6] and Newell [7] were applicable to low-sidelobe arrays. We did this by purposely inducing certain errors in some of the measurements and comparing their measured effect with the effect predicted by theory. We also estimated the total expected uncertainties for this experiment. By comparing the final far field determined from the PNF technique to that determined using the far-field technique, we show that the differences are within what is expected from these analyses.

Finally, we investigated methods of reducing the effects of uncertainties. In particular, we showed that a difference (Δ) probe with a null in the direction of the main beam of the antenna under test (AUT) can be used to reduce the effects of some uncertainties.

### 2.2 Description of the Arrays

**Fig. 1:**
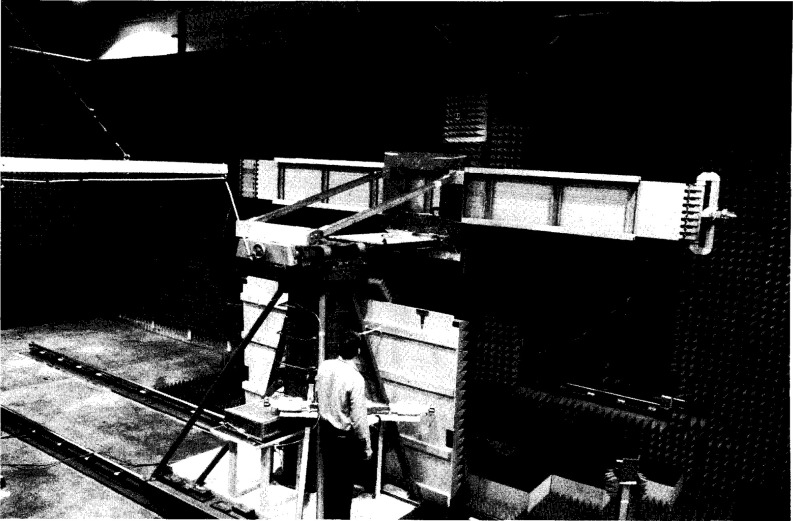
The ULSA array.

An S-band ULSA was used as the primary test antenna for this project. This antenna consists of eight equal-length slotted waveguides, fed at one end by a resonant waveguide manifold and terminated at the other end by high quality matched loads. The waveguide feed manifold has eight identical slotted T-junctions, fed in groups of four from each end of the vertical manifold waveguide which has a short circuit at its center. As illustrated in Fig. 1, the ULSA has a rectangular aperture (4.8 m × 0.4 m), and is designed for a −60 dB Chebyschev azimuth pattern with uniform illumination in elevation.

**Fig. 2:**
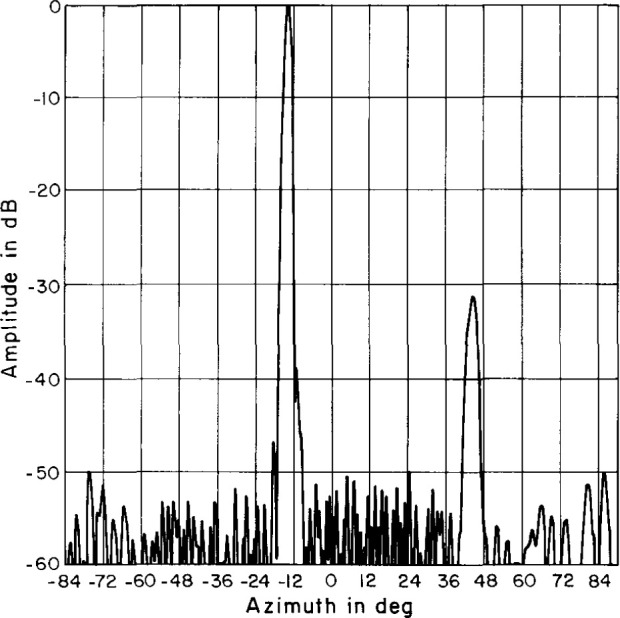
Azimuth plane pattern of the ULSA taken in 1978 at 3.025 GHz.

**Fig. 3:**
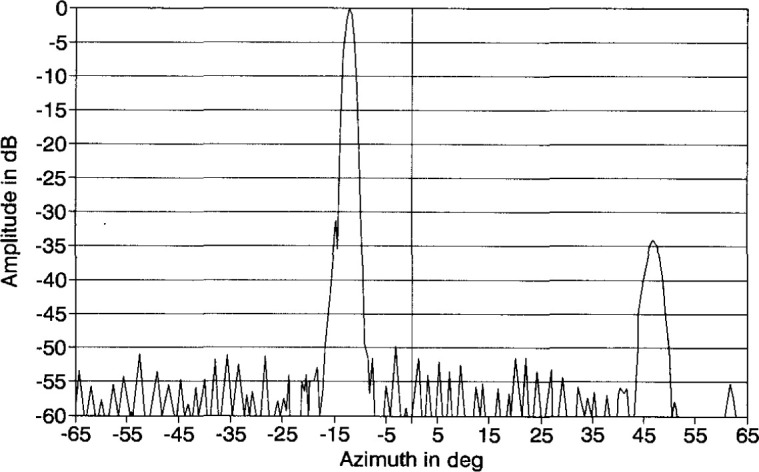
Azimuth plane pattern of the ULSA taken after refurbishment at 3.0 GHz.

An azimuth pattern, measured in 1978 at 3.025 GHz on a far-field range, is shown in Fig. 2. In this pattern, we note three things: (1) the main lobe is squinted off normal (toward the feed end) by an angle of −13°; (2) there is an anomalous lobe at + 44° about 31 dB below the main beam; and (3) the wide-angle sidelobes are well below −50 dB. After the array was refurbished for this project in 1983, the azimuth pattern was measured on the far-field range at 3.0 GHz (Fig. 3). Comparing Fig. 2 with Fig. 3 shows good agreement particularly in terms of the wide-angle sidelobe levels. In 1989, NIST measured the ULSA pattern at 2.9 GHz, 3.0 GHz, and 3.1 GHz. We will discuss these results in Sec. 4.

**Fig. 4:**
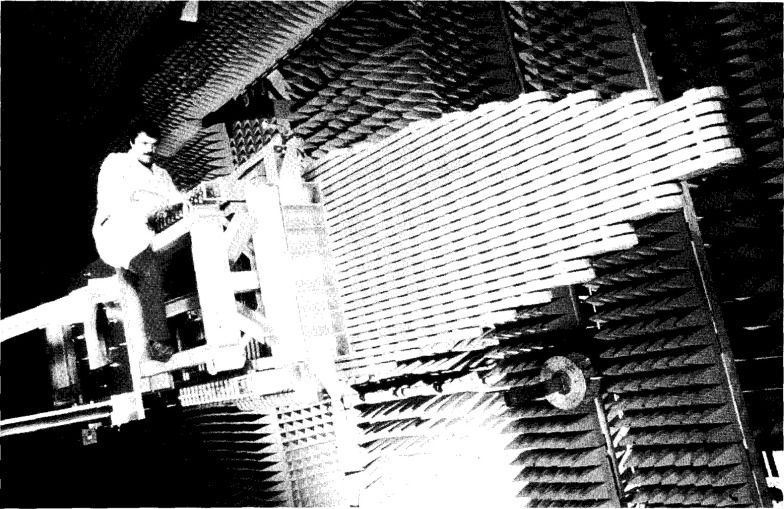
The AWACS array.

The AWACS, which NIST measured in 1984, was built as an engineering prototype model. It has the same aperture dimensions (approximately 7.6 m × 1.5 m) and number of edge-slotted waveguides (28) as the production antennas, but uses manually driven (“trombone”) phase shifters instead of the electrically controlled phase shifters to control the elevation pattern. The sidelobes in the azimuth pattern are actually slightly lower than those finally designed into the production antennas. These patterns cannot be shown because they are classified. However, we performed an analysis on these data and the uncertainties are approximately the same as those for the ULSA measurements. Figure 4 is a photograph of the AWACS array showing it mounted on the NIST PNF range.

The main beam radiated by both the ULSA and the AWACS is not normal to the array face, but is squinted toward the feed end by an angle of [8]

$$\sin\theta =\frac{\lambda }{2S}-\frac{\lambda }{\lambda_{g}},$$ (1)

where λ is the free space wavelength, *S* is the slot spacing, and λ_g_ is the guide wavelength. If we substitute for λ_g_ for the TE_10_ mode using the results of Silver [9], we can rewrite Eq. (1) as

$$\theta =\sin^{-1}\left[\frac{\lambda }{2S}-\sin\left(\cos^{-1}\left(\frac{\lambda }{2a}\right)\right)\right],$$ (2)

where *a* is the inside wide dimension of the waveguide. With this expression, we can predict the squint angles for the ULSA array, using *a* = 7.214 cm and *S* = 5.32 cm, yielding squint angles of 12.7° at 3.0 GHz and 9.7° at 3.1 GHz, respectively. Similarly, we can predict the squint angles of the AWACS using *a* = 5.817 cm and *S* = 4.55 cm.

The near-field scanning tests were then performed for fixed-beam steering directions in accordance with the selected cw test frequency. When we scanned with the Δ probe we steered the probe pattern null to be coincident with the test array squint angle at each test frequency. The use of such a probe and its design is described next.

### 2.3 Design of the Difference Probe

Huddleston has shown that an optimum probe pattern can minimize uncertainties in the measured test antenna spectrum due to truncation of a finite scan plane [10]. The Huddleston probe has a pattern of increasing directivity for conditions of decreasing scan plane area. For a particular planar scanning geometry, the optimum probe concentrates the received power within the available near-field sampling grid; that is,

$$\int \int {\left|v_{L}\left(x,y\right)\right|}^{2}dx\;dy=P_{0}\left(1-{\epsilon^{2}}_{xy}\right),$$ (3)

where *v*_L_(*x,y*) is the scan plane voltage, *P*_0_ is the total power radiated into the forward hemisphere, and *ϵ_xy_* measures the concentration of |*v*_L_(*x,y*)|^2^.

The fractional power not concentrated by the probe over the finite scan area is *P*_0_*ϵ*^2^*_xy_.* Upon transform, the measured coupling product will be an aliased version of the true spectrum. The magnitude of the difference between the aliased and true spectra has a maximum uncertainty of

$$\int_{-k_{x\;\max}}^{k_{x\;\max}}\int_{-k_{y\;\max}}^{k_{y\;\max}}{\left|A_{L}-{A_{L}}^{\prime }\right|}^{2}dk_{x}\;dk_{y}\leq \frac{P_{0}{\epsilon^{2}}_{xy}}{{\left(2\pi \right)}^{2}},$$ (4)

where |*k_x_*_max_| = π/*δ_x_*, *δ_x_* is the *x* sampling increment, |*k_y_*_max_| = π/*δ_y_*, *δ_y_* is the *y* sampling increment, *A*_L_ is the true spectrum, and *A*_L_′ is the measurement estimate of *A*_L_.

Huddleston probes minimize *P*_0_*ϵ*^2^*_xy_* by radiating with higher directivity as the ratio of test antenna area to scan plane area approaches 1. For low-sidelobe antennas, we can extend the optimum probe concept by requiring that Eq. (4) apply only over the test antenna’s sidelobe region. This leads to an expression for a near-field scan voltage, based on a band-limited version of the reaction integral written by Joy, as [11]

$$v\left(x,y\right)=\int_{-\infty }^{\infty }\int_{-\infty }^{\infty }\left[k_{x}A\times \left(Bw\right)\right]e^{-j\;k_{z}z}e^{\left(j\;k_{x}x+j\;k_{y}\right)}dk_{x}\;dk_{y},$$ (5)

where *w* is a spectral windowing function to be imposed on the coupling product spectrum via the probe’s far-field pattern. For usual broad beam open-end waveguide probes, *W*(*k_x_, k_y_*) = 1, and the coupling product spectrum *A* · (*Bw*) is band limited only by the high attenuation of the evanescent modes on the scan plane; that is, *A* · (*Bw*) *→* 0 for *k_z_* ≥ [1*−*(*k_x_*/*k*)^2^ *−* (*k_y_*/*k*)^2^]^0.5^ and |z| > λ.

**Fig. 5:**
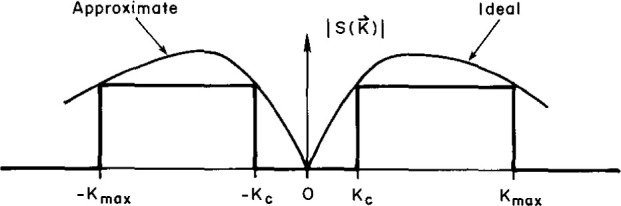
The ideal probe pattern.

However, if *w* is an intentional stop band in the probe pattern (that is, a null region), then the near-field coupling product spectrum will be band limited by both evanescent cutoff as well as real angle filtering. The ideal probe pattern weighting function for sidelobe testing is then given by

$$\begin{array}{c} W\left(k_{x},k_{y}\right)=1\;\text{if}\;k_{c}<\left|k_{x,y}\right|<k_{x\max,\;y\max},\;\text{sidelobe}\\ =0\;\text{if}\;0\leq \left|k_{x,y}\right|\leq k_{c},\;\text{mainbeam} \end{array}$$ (6)

and is sketched in Fig. 5. This pattern is not realizable for any practical small aperture probe, but a useful approximation has been designed and built by combining the anti-phased outputs of two collinear waveguide elements. This probe has been called the difference or Δ probe. In-phase combining of elements produces a sum or Σ probe.

**Fig. 6:**
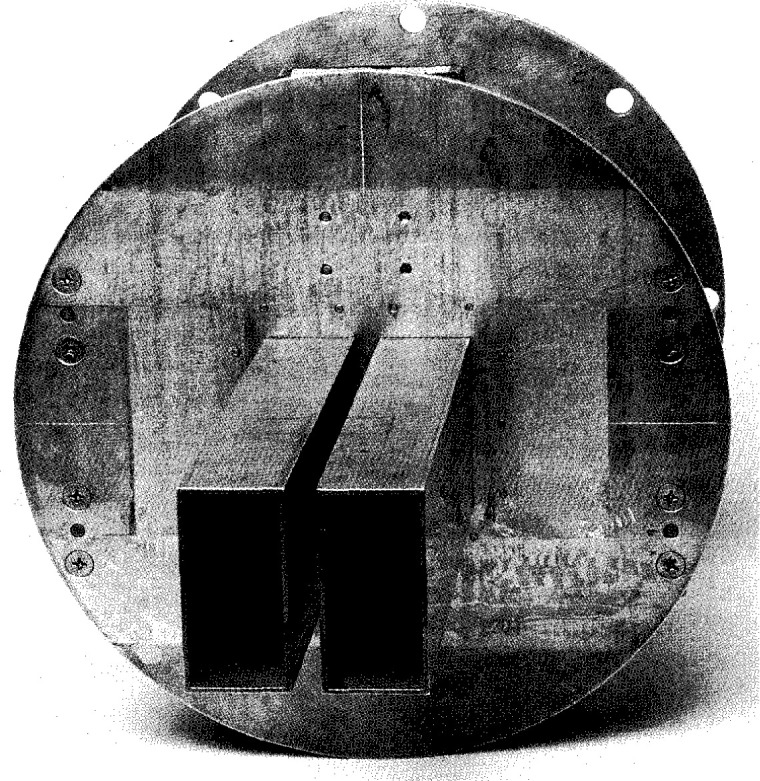
The Δ probe showing both its elements.

**Fig. 7:**
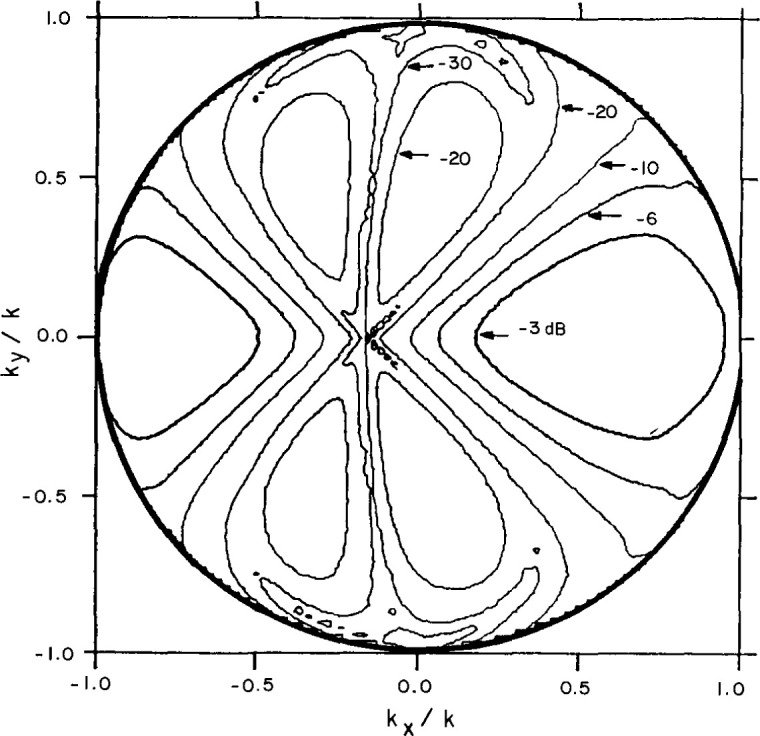
Contour plot of the Δ probe azimuth pattern.

Figure 6 shows one of three two-element probes built for this project, and Fig. 7 shows a contour plot of this probe’s measured far-field azimuth pattern at 3.1 GHz. The shifted null is accomplished by physically steering the Δ-probe by an appropriate angle or by using unequal cable lengths between the transition of each waveguide element and an integral coaxial hybrid coupler mounted on the probe carrier plate. The waveguide elements for the probe of Fig. 6 were spaced horizontally by 4.8 cm (0.5 λ at 3.1 GHz). The unequal length cables to the hybrid combiner provided a fixed null shift coincident with the test array’s mainbeam pointing direction. More commonly, the null coincidence was maintained by mechanically rotating the probe carrier plate to fixed null pointing directions and then using equal length cables to the hybrid. Of course, we must measure two-dimensional vector probe patterns for all probe-frequency combinations prior to near-field scanning.

### 2.4 Description of the Measurement Facility

A typical near-field measurement system can be conveniently discussed in terms of three subsystems: (1) computer, (2) rf source and receiver, and (3) mechanical scanner and probe positioner. A great deal of variety is possible for each of these subsystems. We will describe only the essential features of each subsystem used at the NIST PNF measurement facility.

#### 2.4.1 Computer

Because of the large amounts of data involved, computer control of the measurement system is essential. In the NIST measurement facility, a special purpose microcomputer is used as the position controller. The position controller receives digital inputs from the *x–y* position encoders (in this case a laser interferometer), controls the motor drives moving the probe, and triggers the receiver to perform measurements at predetermined points. The NIST facility also has a minicomputer which records the data on a data storage device, (either magnetic tape or hard disk), monitors the receiver and position controller for errors, and performs some of the data analysis during the diagnostic tests. A mainframe computer (1983) or 386 PC (1989) is used for the bulk of the data analysis, especially the two-dimensional FFT.

#### 2.4.2 RF System

**Fig. 8:**
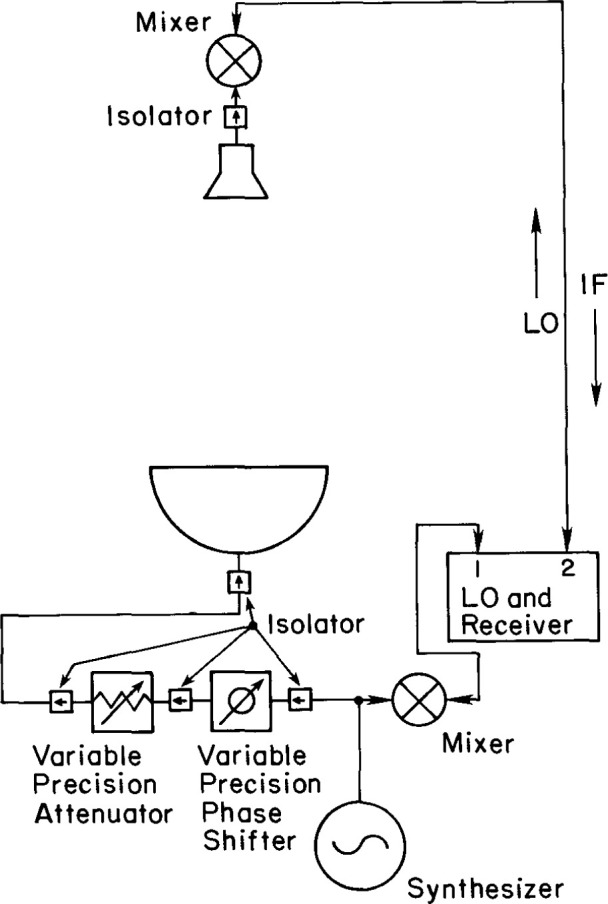
Rf measurement system schematic.

The basic components of the rf system are shown schematically in Fig. 8. They are: the transmitting and receiving antennas, isolators, mixers, variable attenuator and phase shifter, receiver, and synthesizer (signal source). The signal source must be stable in frequency and power level to minimize its contribution to measurement uncertainty. Generally, a frequency stability of a few parts in 10^6^ and amplitude variations of less than 0.1 dB over the total scan time are adequate. Tie scans can be used to correct for small amounts of drift which are on the order of a few degrees in phase and a few percent in amplitude over the total scan time. The source power output requirements depend on the gains of the AUT and the probe as well as the mixer sensitivity. The maximum magnitude of the near-field amplitude |*b*_0_′(*P*)|_max_ is approximately given by

$${{\left|b_{0}^{\prime }\right|}^{2}}_{\max}\approx *\left(G_{p}/G_{a}\right){\left|a_{0}\right|}^{2},$$ (7)

where *G*_p_ and *G*_a_ are respectively the probe and AUT gains, and *a*_0_is the input amplitude to the transmitter. To reduce noise for the low-sidelobe measurements, *b*_0_′(*P*)|_max_ should be at least 65–70 dB above the noise but also be low enough to be within the linear range of the mixer. When the probe is cross polarized to the AUT, the maximum signal drops to about 30 dB below the maximum signal of the co-polarized case. It is desirable for the cross-polarized maximum signal to be of the same order as the co-polarized maximum signal. This can be achieved by increasing *a*_0_ by increasing the receiver gain, or by some combination of the two.

A critical requirement of the source, and in fact for the complete rf system, is that it be well shielded. Signal leakage from the source, transmission lines, and input components, or signal pick-up by similar parts of the receiving system can cause significant measurement uncertainties in the near-field amplitude and phase. Tests must be performed to guarantee that the whole system is well shielded and that signals can only be transmitted and received via the AUT-probe transmission path.

The receiver is a very important part of the measurement system and must accurately measure the near-field amplitude and phase of the rf signal over a dynamic range of at least 80 dB. It must be stable over the time required for near-field scanning, which may be hours, and have good linearity and resolution in its conversion from rf signal to digital output.

#### 2.4.3 Mechanical Scanner and Probe

**Fig. 9:**
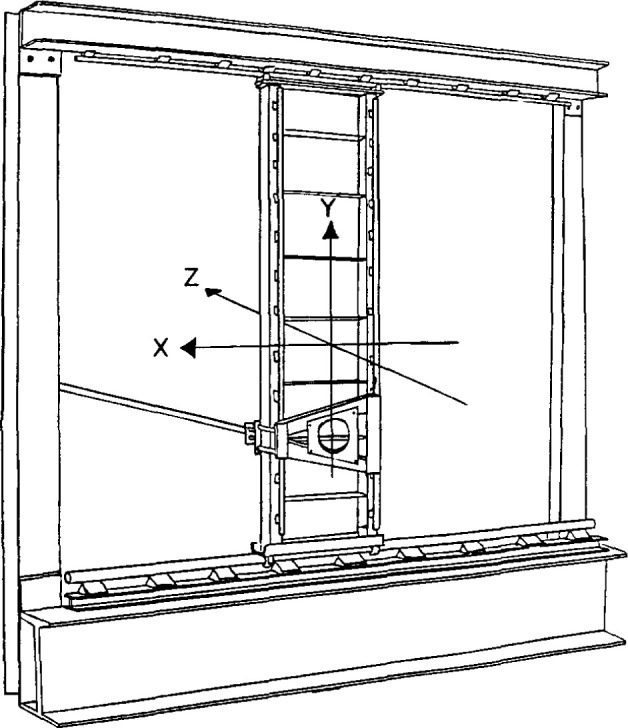
Box frame planar scanner.

The mechanical scanner or probe positioner consists of the supports, guides and drive motors to move the probe over the planar area, the encoders to measure the probe position, and the rf transmission line to couple the probe output to the receiver. The “box frame” design of the NIST scanner, Fig. 9, was one of the first PNF range designs. A large, rigid frame, constructed from metal I beams, serves as the base and support for the two horizontal guide rails. Both rails are precision ground, stainless steel cylinders supported at intervals of about 30 cm. Precision linear ball bearings attach the vertical column to the horizontal guide rails. The box frame requires additional support to maintain a rigid vertical structure. This is accomplished by attaching it to a stable interior wall.

**Fig. 10:**
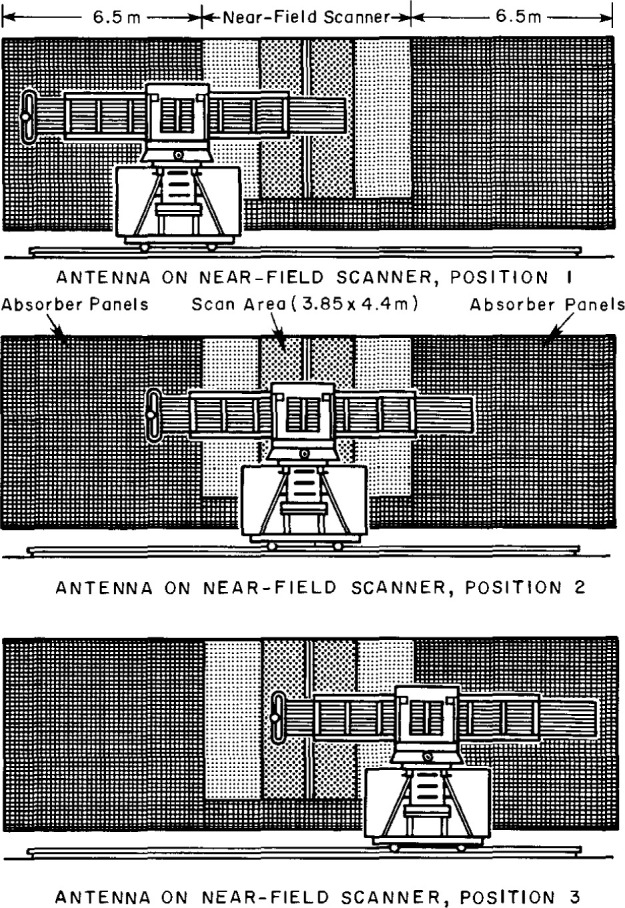
Schematic of antenna in three positions relative to the scanner.

At the NIST facility an auxiliary linear translator has been attached to the AUT to extend the effective measurement area, as shown in Fig. 10. The essential requirement of this linear translator is that the translation be precisely known and controlled to correctly combine different segments of the measurement plane. Also, to correctly combine these different segments there should be some overlap between them. By comparing the amplitude and phase of the overlap regions of the different segments, we can correct for both amplitude and phase drift between segments.

A common concern in any scanner is to ensure that the rf transmission line between the probe and receiver move without causing significant amplitude or phase change in the measured data. At the NIST facility, the transmission line is supported on a scissors-arm mechanism so that rotation is of concern at only three joints. We use service loops of semirigid or flexible coaxial cable at these three points to produce a stable transmission line.

## 3. Preliminary Tests

Several preliminary, one-dimensional tests are performed to determine the required near-field spacing, required scan size, the best value of the probe-antenna separation distance, and to determine the level of the leakage and reduce it if necessary. These tests can also be used to estimate some of the measurement uncertainties. However, two-dimensional tests (described in Sec. 4) provide better uncertainty estimates since one-dimensional tests assume that the pattern is separable into functions of *X* and *Y.* Usually, this is only approximately true.

### 3.1 Test Space

The test space procedure is used to determine the required near-field spacing. It consists of taking one-dimensional scans in *X* and *Y* with very fine spacing (≈ 0.05 λ). First an FFT is performed on the full set of data, then using only every other point, then using every third point, and so forth. From this, the far-field spectra from various spacings can be compared. The smallest spacing is assumed adequate and when the spacing from the other FFTs is so large that the spectrum changes by more than the desired accuracy a spacing equal to or smaller than the next smallest spacing is chosen.

From these one-dimensional tests we concluded that the spacing between near-field data points should be about 0.4 λ_min_ in both *X* and *Y*, where λ_min_ is the wavelength at the highest measurement frequency.

### 3.2 Test Scan

The test scan procedure is similar to the test space procedure in that one-dimensional centerline scans in *X* and *Y* are performed. In this case, very long centerline scans are performed, and data are truncated from the edge in various amounts. As in the test space procedure the computed spectra are compared and a required scan length determined. The choice of scan area was 10.4 m in *X* by 3.8 m in *Y.*

### 3.3 Multiple Reflection Tests

A *Z*-multiple-reflection test, where data are taken as a function of Z at several fixed *X* and *Y* values, is performed. A separation distance is chosen to minimize the peak-to-peak variations as a function of *Z*. From these tests the probe-antenna separation distance was chosen to be 65 cm for the ULSA and 35 cm for the AWACS.

**Fig. 11:**
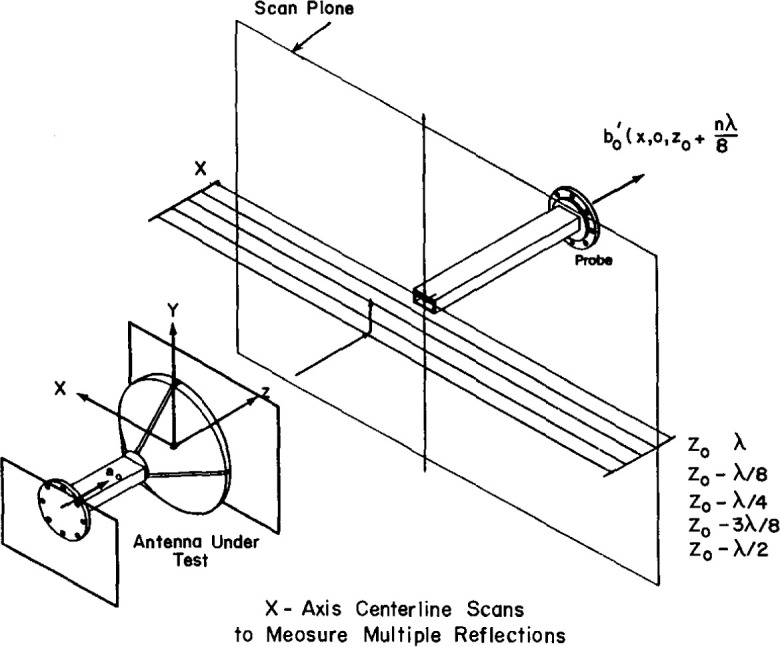
Schematic of centerline multiple reflection tests.

A second multiple-reflection test is designed to estimate the effects on the far field of multiple reflection interactions between the probe and AUT. The test is implemented by taking centerline scans in both *X* and *Y* at several different separation distances (see Fig. 11). Consecutive separation distances differ by λ/8. One of these separation distances is equal to the separation distance determined using the first *Z*-multiple-reflection test.

An FFT is performed on the centerline scans to obtain a one-dimensional far field; these far fields are averaged together after correcting the phases for the different separation distances. This average is then subtracted in a complex manner from each one-dimensional centerline-scan far field to estimate the magnitude and character of the multiple reflections. Additionally, the average far field can be transformed back to the near field to obtain an average one-dimensional near field. This average can be subtracted from the individual centerline near-field scans to obtain the near-field character of the multiple reflections. The gain and one-dimensional patterns can be compared and the uncertainty due to multiple reflections estimated.

**Fig. 12:**
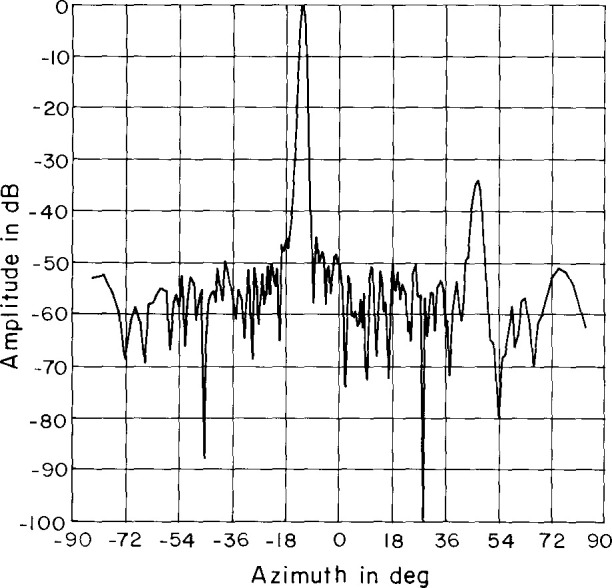
Sample result for the far field after averaging for multiple reflections.

**Fig. 13:**
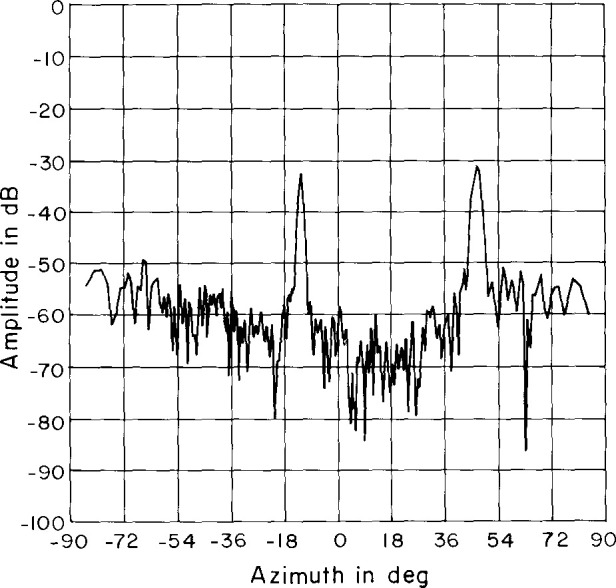
Sample multiple reflection spectrum amplitude, using the sum probe, relative to the peak average far field for the sum probe.

**Fig. 14:**
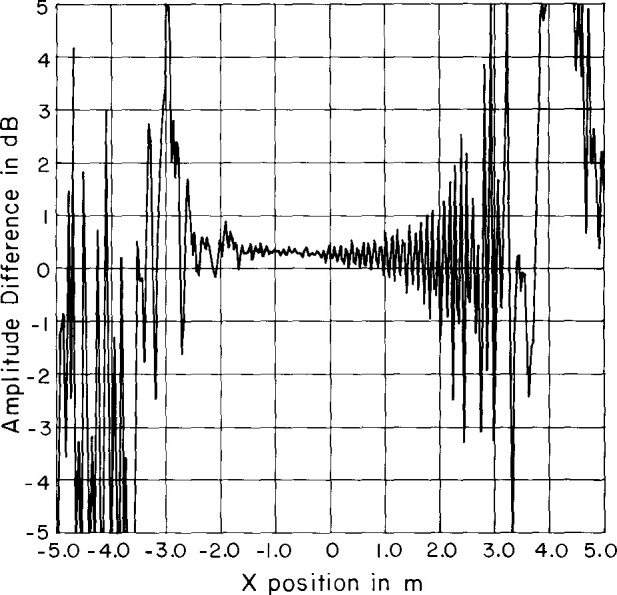
Sample multiple reflection near-field amplitude using the sum probe.

**Fig. 15:**
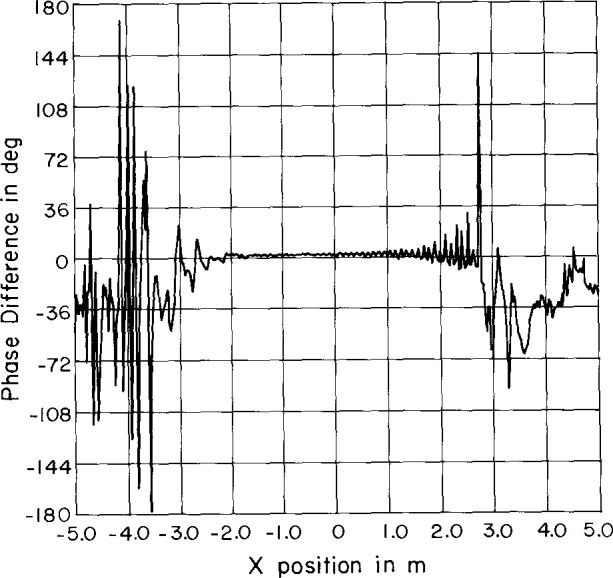
Sample reflection near-field phase using the sum probe.

**Fig. 16:**
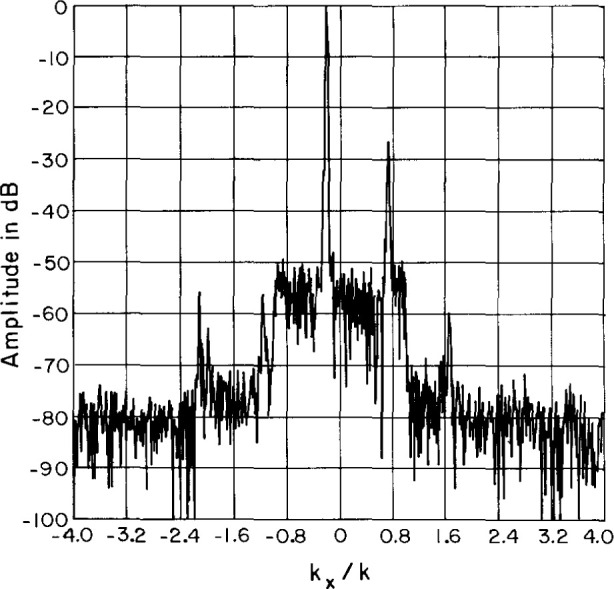
Test space far-field spectrum showing multiple reflection lobes.

Sample results are shown in Figs. 12–15. Surprisingly, the character of the multiple reflections bears some resemblance to the average far-field pattern. We also find that there are two different periods in the near-field amplitude and phase multiple reflections. The predominant period is twice the spacing between elements and corresponds to the spacing between elements of the same slant direction (every other element). This period is clearly visible in Figs. 14 and 15. The second period corresponds to the spacing between consecutive elements and only becomes apparent in the invisible space part of the far-field spectrum (Fig. 16). Each period produces distinctive lobes in the computed spectrum. The location of each lobe depends on the corresponding periodicity in the near field. The first lobe is due to a near-field periodicity of approximately λ and occurs in the computed spectrum at |*k_x_*/*k| ≈* 1 (relative to the main beam direction) or near *θ* = 90°. The second lobe is due to a near-field periodicity of approximately λ/2 and therefore occurs in the computed spectrum at |*k_x_*/*k| ≈* 2 (relative to the main beam direction). This is in the invisible part of the spectrum.

### 3.4 Leakage Tests

Leakage tests are performed by taking centerline scans with a termination on the receiving side in place of the probe to test for leakage on the receiving side and by doing centerline scans with a termination on the transmitting side in place of the AUT. The significant sources of leakage were located and shielded so as to reduce the leakage level to −75 dB relative to the near-field peak.

## 4. Two-Dimensional Measurement Results

### 4.1 Summary of Measurements

Some two-dimensional near-field measurements were performed with each of the probes. In order to compare the results and the uncertainties associated with each probe there was some overlap in these measurements. These measurements are summarized in Table 1.

Fewer measurements were performed with the Σ probe. This was because after a few measurements this probe was found to behave in a manner closely resembling the open-end waveguide.

Measurements were performed at different distances to estimate the effects of multiple reflections between the probe and the AUT.

### 4.2 Combining Segments

Both the ULSA and the AWACS have long dimensions which exceed the capability of the NIST scanner. It is, therefore, necessary to measure the near field of both these antennas in segments and to combine these segments into one near field. As discussed earlier, the purpose of the linear rails is to allow the AUT to be moved while preserving an accurate knowledge of the *X*-position.

In order to combine the three segments accurately we must account for the effects of both amplitude and phase drift. This is accomplished by requiring some overlap between adjacent segments. In particular, the measurement was performed with adjacent segments having an overlap of five scans which were taken vertically.

A computer program, STITCH, was used to combine adjacent segments using the following technique. The amplitude and phase of the center section which contained the near-field peak signal was used as the reference. The amplitudes and phases of the adjacent segments were compared to those of the center segment in the overlap region. This comparison was done by computing an amplitude ratio and phase difference at each point in the overlap region. These amplitude ratios and phase differences were averaged using a weighting which was proportional to the square of the amplitude. The amplitudes of each point in the adjacent segments were corrected by multiplying by the average of the ratio of the center segment amplitude to the adjacent segment amplitude. The phases were corrected by adding the average phase difference.

**Fig. 17:**
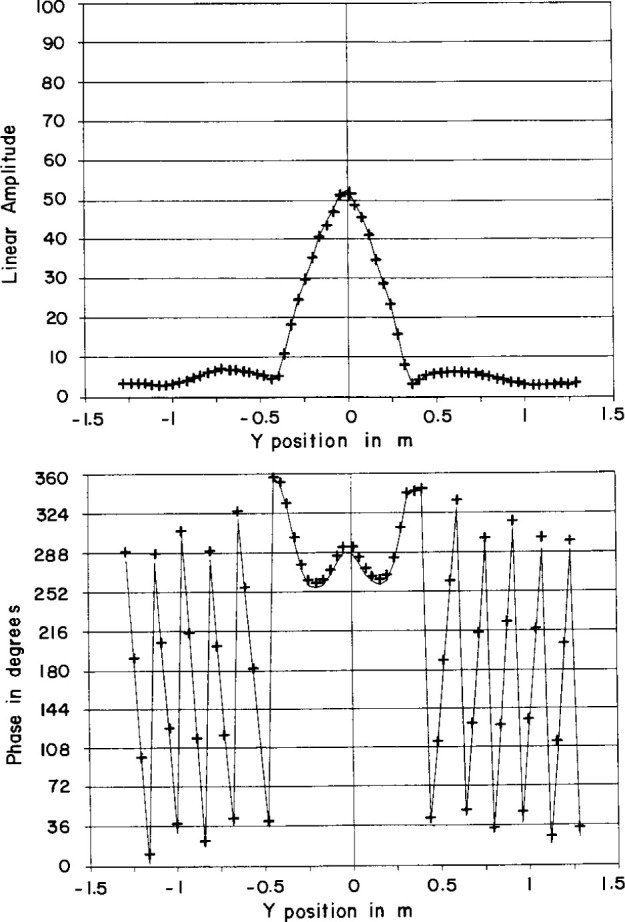
A comparison of two vertical scans from adjacent segments which overlap, amplitude (top), phase (bottom), scan 1 (solid line), scan 2 (+ + +).

The amplitude correction was a few percent at most and the phase correction was a few degrees at most. A comparison of two typical overlap scans is shown in Fig. 17.

### 4.3 Room Scattering Test

To estimate the effects of room scattering NIST developed a room scattering test, SCAT, which takes advantage of the unique feature of the NIST facility of being able to move both the AUT and the probe in the *X*-direction.

Ideally, to measure room effects one would like to move the room while keeping the antennas fixed. This is obviously impractical! Instead, we set up a test procedure where the antenna and probe were moved together in the *X*-direction relative to the room so that the *X*-coordinate of the probe with respect to the AUT was fixed. A *Y*-scan was taken at each *X*-position. These scans would be identical if there were no room scattering. The room scattering can be estimated by obtaining a single average *Y*-scan and subtracting it from each of the others; an FFT is then performed on the resulting two-dimensional data to obtain an estimate of the room-scattering far-field spectrum.

**Fig. 18:**
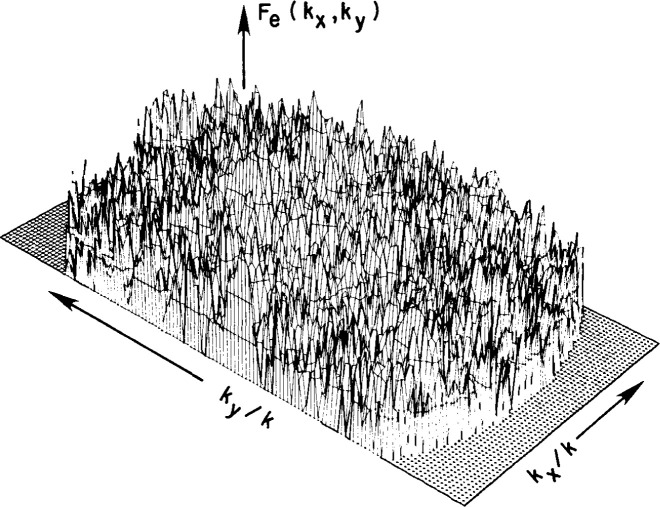
Sample scattering far field using the open-end waveguide. Peak is −70 dB relative to the peak AUT far field.

The results of the tests indicated that the room-scattering far field was approximately random (see Fig. 18) and the peak value was about −70 dB relative to the peak of the AUT’s far field. The root mean square (RMS) value of the room scattering was found to be −89 dB for the open-end waveguide and −94 dB for the Δ probe.

### 4.4 Truncation Tests

**Fig. 19:**
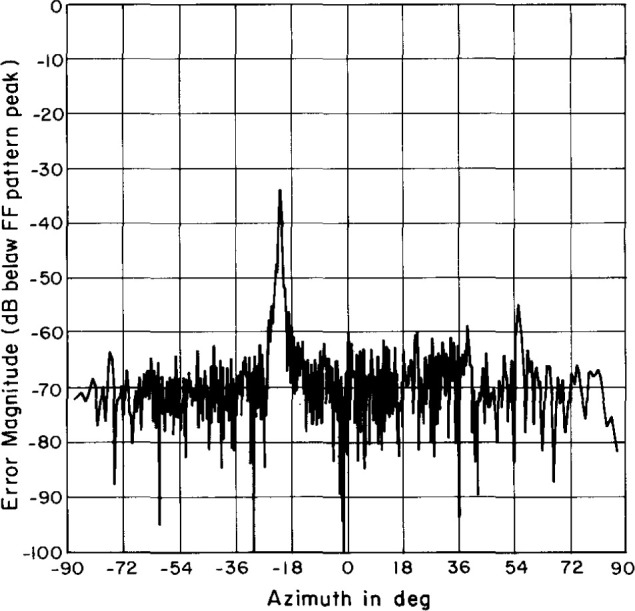
Azimuth cut of the truncation spectrum using the open-end waveguide probe.

**Fig. 20:**
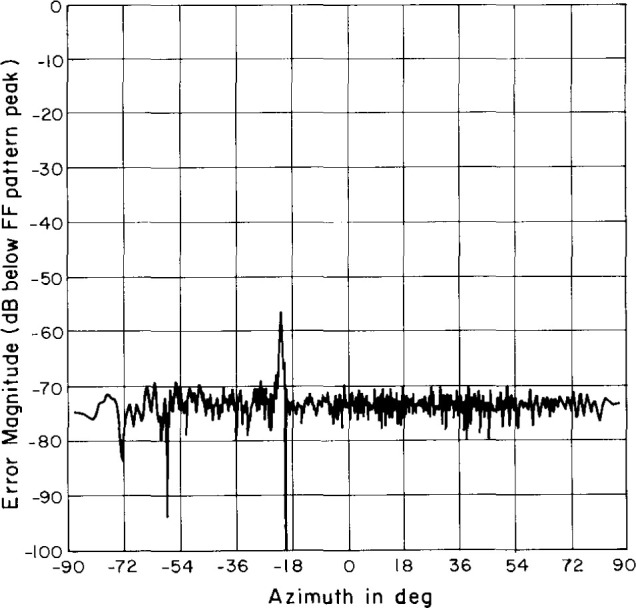
Azimuth cut of the truncation spectrum using the Δ probe.

According to Yaghjian [6] we can estimate an upperbound to the uncertainty due to scan plane truncation by performing an integration of the data along the outside edge of the near-field scan area. We obtained this spectrum by setting the rest of the near-field data to zero and performing the usual FFT. The resulting truncation spectra for the azimuth cut are shown in Fig. 19 (for the open-end waveguide – AWACS combination) and Fig. 20 (for the Δ probe–AWACS combination). These plots are normalized relative to the AUT’s peak far field. The Δ probe is on the whole about 7 dB better than the open-end waveguide. The magnitude of the truncation spectrum is generally between about 63 dB and 70 dB below the far-field peak for the open-end waveguide and between about 70 dB and 75 dB below the far-field peak for the Δ probe.

### 4.5 Simulated Position Error

To verify the analysis of Yaghjian [6] and Newell [7] with respect to position errors some measurements were done to simulate the effects of periodic *Z*-position errors. The analysis predicts that *Z*-position errors have a smaller effect on the spectrum obtained using the Δ probe than on the spectrum obtained using the Σ probe or open-end waveguide. Four PNF measurement scans were done for this simulation, two for the Σ probe and two for the Δ probe. Of the two scans for each probe, one had no errors introduced and the other had periodic errors purposely introduced. These errors had periods of 2.5 λ, 1.67 λ, and 1.11 λ. The magnitude of the error for each period can be found in Table 2. In addition, Table 2 contains a summary of the spectral location, predicted spectral error, and the actual measured errors.

**Fig. 21:**
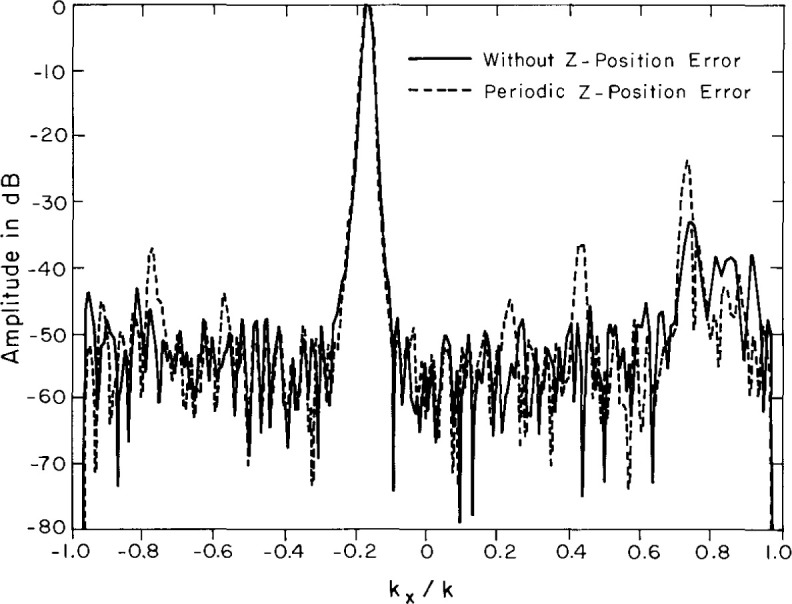
Azimuth far-field cut for the sum probe with simulated position errors (dashed line) and without errors (solid line).

**Fig. 22:**
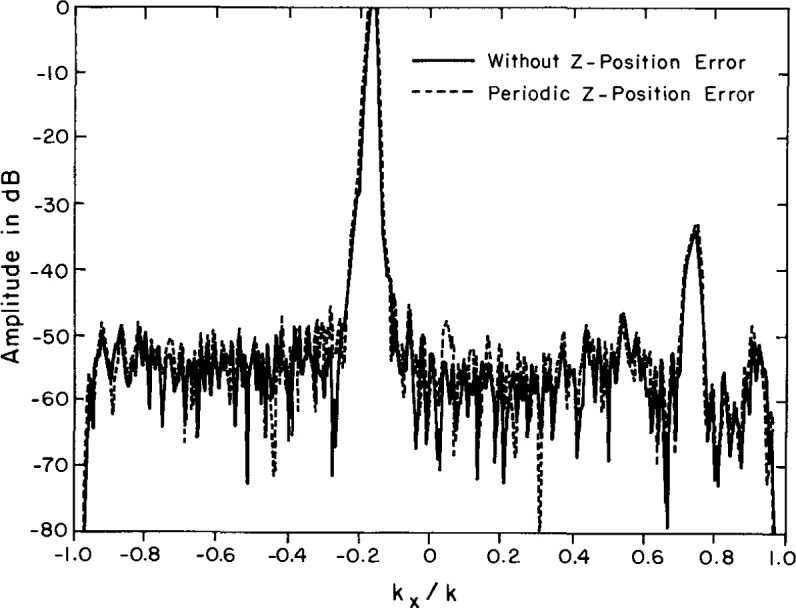
Azimuth far-field cut for the Δ probe with simulated position errors (dashed line) and without errors (solid line).

Table 2 shows that the measured errors are in substantial agreement with the predicted. More important, as predicted by theory, the simulated position error has a smaller effect on the spectrum obtained using the difference probe than on the spectrum obtained from the Σ probe. Figure 21 compares the far field with and without errors for the Σ probe, and Fig. 22 is the same comparison for the Δ probe. As expected the position errors are smaller for the Δ probe than for the Σ probe.

### 4.6 Probe-AUT Multiple Reflections

We can obtain a better estimate of the uncertainty due to probe-AUT multiple reflections by taking a full set of two-dimensional near-field data at two different separation distances which differ by λ/4. Then we average (in a complex manner) the two far-field spectra and subtract (again in a complex manner) the average from each of the individual spectra to obtain a multiple-reflection spectrum.

**Fig. 23:**
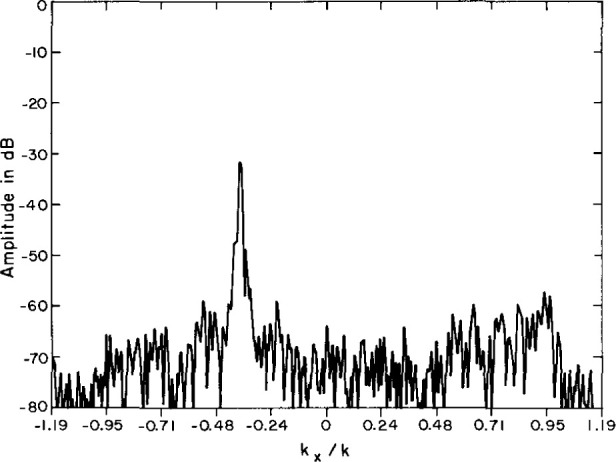
Multiple reflection spectrum for the waveguide probe using data taken from two near-field scans separated by λ/4.

**Fig. 24:**
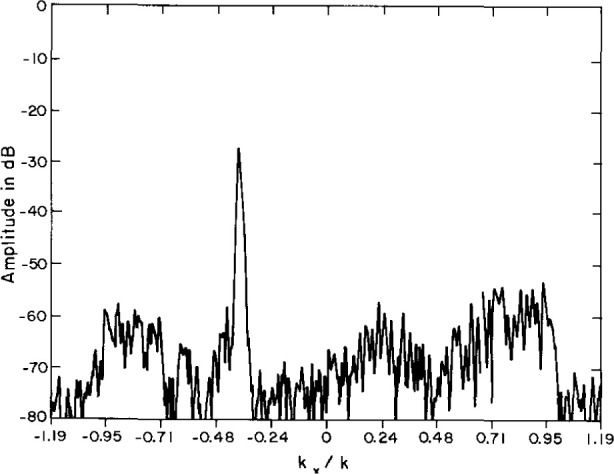
Multiple reflection spectrum for the Δ probe using data taken from two near-field scans separated by λ/4.

The resulting spectrum for the open-end waveguide-AWACS combination is shown in Fig. 23 and that for the Δ probe and AW ACS is shown in Fig. 24. The multiple-reflection spectrum for the Δ probe is 3 dB to 5 dB higher than that for the waveguide probe. The multiple reflections associated with the Δ probe are greater because the Δ probe is larger.

### 4.7 Data Processing

For an arbitrarily polarized test antenna, the transmitting coefficient for the main and cross polarizations are given by Eq. (8a) and Eq. (8b). Since the D′ and D″ terms are scalar quantities, the measured far-field polarization of the test antenna will be determined by the polarization components of the probe calibration files. If the probe and test antenna have matched main-polarized components and the cross-polarized responses of the probe and test antenna are small, we then use Eq. (10a) to obtain the main component of the test antenna.

**Fig. 25:**
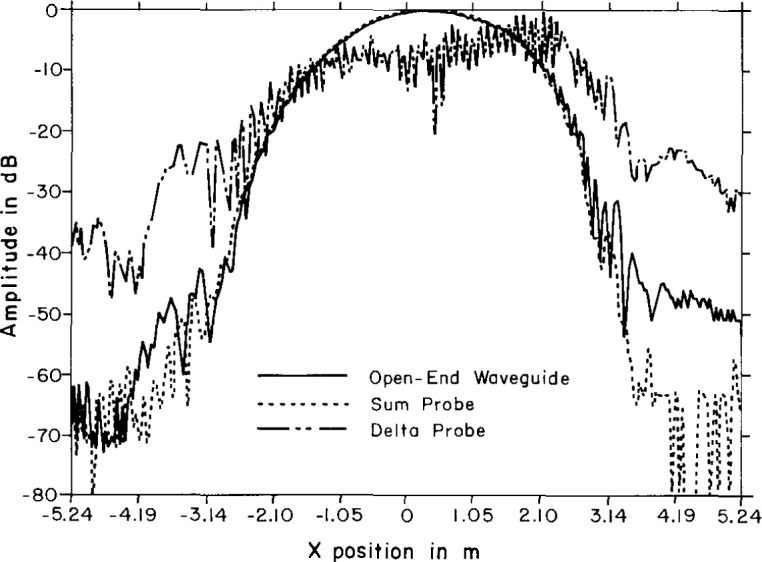
Near-field centerline cut for the amplitude using the Δ probe (dashed line), the sum probe (dotted line), and waveguide probe (solid line).

**Fig. 26:**
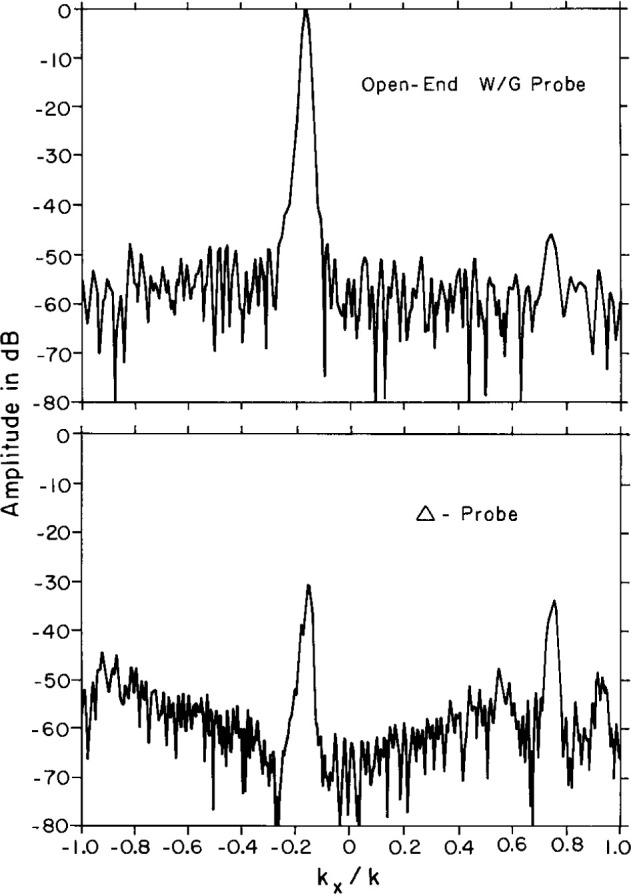
Centerline coupling product amplitude using the waveguide probe (top) and using the Δ probe (bottom).

**Fig. 27:**
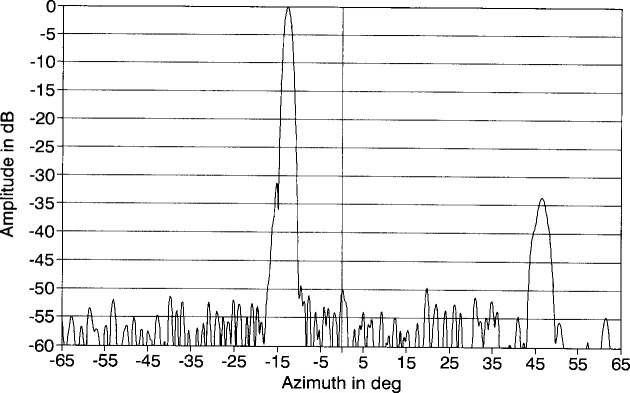
Probe corrected centerline far-field pattern for the ULSA.

Near-field centerline amplitude scans for the ULSA array as measured by the Δ probe, the Σ probe, and the open-end waveguide for a *Z*-separation of 65 cm are shown in Fig. 25. The filtering effect of the Δ probe is responsible for the compression of the near-field dynamic range as well as much of the high-frequency amplitude ripple. Following FFT processing, we show the corresponding coupling product transforms for the open-end waveguide and the Δ probe in Fig. 26, plotted over the real wavenumber space only (|*k_x_*/*k*| < 1.0). The coupling product for the Σ probe differs little from that of the waveguide probe and we do not show it here. The filtering property of the Δ probe is clearly visible by noting the reduced main beam and suppressed sidelobes near the main beam. Similarly, the Σ probe filters the sidelobes at wide angles beyond the large anomalous lobe (|*k_x_*/*k*| >0.8) but is accurately measuring the mainbeam region. The coupling product spectrum can be probe-corrected to obtain the principal azimuth angle pattern of the ULSA array. Figure 27 shows the resulting probe-corrected far field for the waveguide probe at 3.0 GHz.

To certify the measurement uncertainty due to all random sources, two-dimensional evanescent scanning tests were conducted with the AWACS array. Near-field spacings of 3.81 cm ensured that the coupling product spectrum would extend to (|*k_x_*/*k*|, |*k_y_*/*k*|) = 1.19 at the selected test frequency, thus exposing the so-called evanescent spectrum whenever [(*k_x_*/*k*)^2^ *+* (*k_y_*/*k*)^2^]^0.5^*≥* 1. Because all evanescent antenna modes actually radiated by the test antenna are attenuated way below the dynamic range of the PNF instrumentation only a short distance from the antenna, we do not expect to intercept antenna evanescence during scanning with any probe at Z = 35 cm (3.85 λ). Therefore, the magnitude of the coupling-product spectrum in this evanescent region is a direct measure of the far-field noise power.

**Fig. 28:**
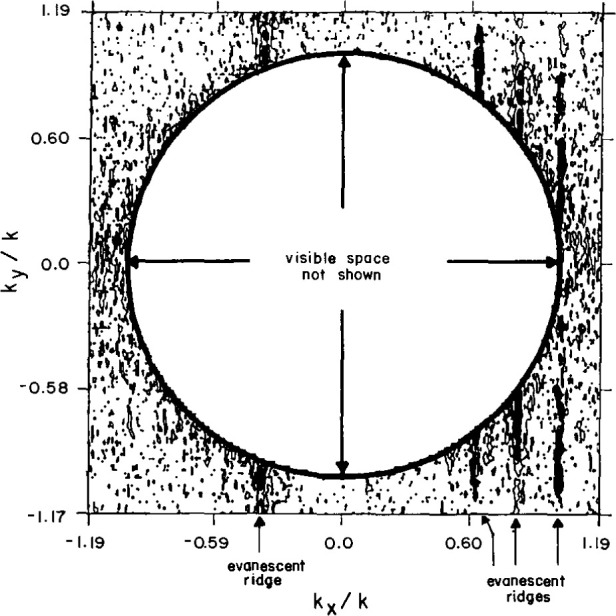
Evanescent spectrum contour plot using the open-end waveguide. Note the ridges at *K_x_*/*K* = −0.37, 0.64, 0.82, 1.02.

**Fig. 29:**
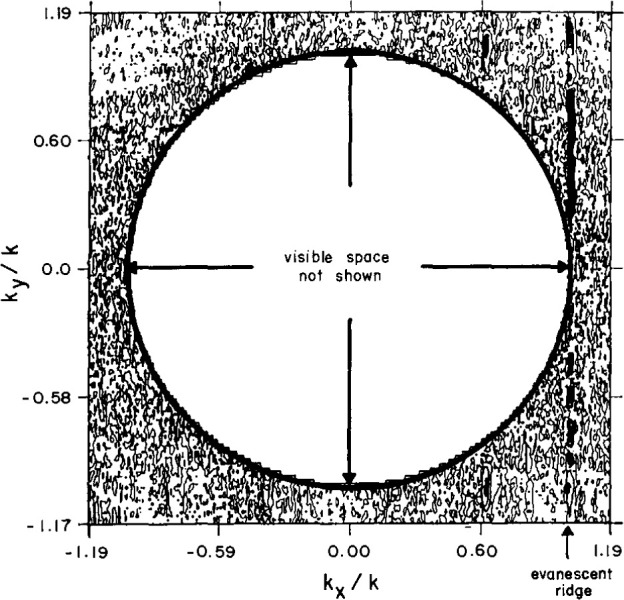
Evanescent spectrum contour plot using the Δ probe. Note the ridge at *K_x_*/*K* = 1.02.

These evanescent spectra generally show a random distribution of sidelobe peaks in the region beyond the visible space limits, at or below −80 dB for the open-end waveguide probe. For the Δ probe the spectrum is normalized to the peak of the open-end waveguide probe; the evanescent sidelobes have random peaks below −90 dB. However, in both spectra, the evanescent spectra also contain distinct sidelobe ridges at about −55 dB (Figs. 28 and 29), which cannot actually be radiated because, of course, the evanescent PNF are too highly attenuated to be measured at *Z* = 35 cm. Therefore, these evanescent sidelobe ridges must be the result of undiagnosed periodic scan-plane error–most probably due to unavoidable periodic multipath interactions. By excluding these ridges, we may compute the RMS over all remaining evanescent wavenumbers, and compare this spectral noise average to the peak coupling product mainbeam response. Table 3 contains these measured noise ratios for the AWACS test array. The table shows that the Δ probe AWACS signal-to-noise ratios are better by 12 dB when compared to scanning with a standard open-end waveguide probe.

## 5. Measurement Accuracy

We used the mathematical analyses of Yaghjian [6] and Newell [7] to estimate the uncertainty for these measurements. These analyses allow us to estimate upperbound uncertainties for most of the possible uncertainty sources of Table 4 (from Table I in Newell [7]).

The sources of uncertainty listed in Table 4 fall into two broad categories. The first is uncertainties in the probe parameters arising from the measurements of the probe’s gain, polarization, and pattern; second, uncertainties in the calculated spectra due to uncertainties in the measured near-field data and various reflection coefficients.

### 5.1 Probe-Parameter Uncertainties

Newell [7] has shown that the antenna transmitting coefficients, *t*_m_ (main component) and *t*_c_ (cross component), are given by

$$t_{m}\left(K\right)=\frac{\frac{D^{\prime }\left(K\right)}{s_{m}^{\prime }\left(K\right)}-\frac{D^{\prime \prime }\left(K\right)}{s_{c}^{\prime \prime }\left(K\right)}\rho_{s}^{\prime }\left(K\right)}{1-\frac{\rho_{s}^{\prime }\left(K\right)}{\rho_{s}^{\prime \prime }\left(K\right)}}$$ (8a)

$$t_{c}\left(K\right)=\frac{\frac{D^{\prime \prime }\left(K\right)}{s_{c}^{\prime \prime }\left(K\right)}-\frac{D^{\prime }\left(K\right)}{s_{m}^{\prime }\left(K\right)\rho_{s}^{\prime \prime }\left(K\right)}}{1-\frac{\rho_{s}^{\prime }\left(K\right)}{\rho_{s}^{\prime \prime }\left(K\right)}}$$ (8b)

where *D*′ is the uncorrected (for the probes) far field using probe 1 whose main component is in the same sense as the main component of the AUT, D″ is the uncorrected far field using probe 2 whose main component is in the same sense as the cross component of the AUT, ***K*** is the transverse part of the wave vector, and *ρ*′ and *ρ*″ are the polarization ratios of probe 1 and probe 2 respectively. Hereafter we will drop the explicit use of ***K***. With these conventions, when

$$\left|\rho_{s}^{\prime }/\rho_{s}^{\prime \prime }\right|\;⩽\;1$$ (9a)

and when

$$\left|\rho_{s}^{\prime }/p_{t}\right|\;⩽\;1,$$ (9b)

where *p*_t_ *= t_c_*/*t*_m_, the probe correction equations become [7]

$$t_{m}=\frac{D^{\prime }}{s_{m}^{\prime }}$$ (10a)

$$t_{c}=\frac{D^{\prime \prime }}{s_{c}^{\prime \prime }}-\frac{D^{\prime }}{s_{m}^{\prime }\rho_{s}^{\prime \prime }}$$ (10b)

When the conditions of Eq. (10a) and (10b) apply, we can calculate the differential of these equations to obtain equations for the fractional uncertainties. We find that the fractional uncertainties for *t*_m_ and *t*_c_ are [7]

$$\frac{dt_{m}}{t_{m}}=\frac{dD^{\prime }}{D^{\prime }}-\frac{ds_{m}^{\prime }}{s_{m}^{\prime }},$$ (11a)

$$\begin{array}{l} \frac{dt_{c}}{t_{c}}=\left(1+\frac{1}{p_{t}{\rho^{\prime \prime }}_{s}}\right)\left(\frac{dD^{\prime \prime }}{D^{\prime \prime }}-\frac{{ds^{\prime \prime }}_{c}}{s_{c}^{\prime \prime }}\right)\\ +\left(\frac{1}{p_{t}\rho_{s}^{\prime \prime }}\right)\left(\frac{d\rho_{s}^{\prime \prime }}{\rho_{s}^{\prime \prime }}-\frac{dD}{D^{\prime }}+\frac{ds_{m}^{\prime }}{s_{m}^{\prime }}\right). \end{array}$$ (11b)

The uncertainties in the far field are caused by uncertainties in the values of *D′, D*″, $s_{m}^{\prime }$, $s_{c}^{\prime \prime }$, and $\rho_{s}^{\prime \prime }$. When Eq. (9a) and Eq. (9b) are valid, the polarization ratio of probe 1 $\rho_{s}^{\prime }$, has no significant effect on either component of the far field. In addition, the probe’s effect on the AUT cross component depends on the relative polarization ratios of the AUT and probe 2. Since the main-component uncertainties are proportional to the uncertainties in $s_{m}^{\prime }$, the uncertainties in the probe gain and pattern have a one-to-one correspondence to uncertainties in the AUT’s main component. Typical uncertainties for the probes are given in Table 5.

If the sum probe or open-end waveguide is used to measure the AUT, we expect a standard uncertainty due to the probe of about 0.06 dB in the gain of the AUT. The difference probe, on the other hand, has a 30 dB null in the direction of the AUT main beam; therefore the standard uncertainty in its gain in the direction of the null is about 0.9 dB. In addition, Δ probe steering uncertainties introduce additional gain uncertainties. Because of the sharpness of the null, the steering uncertainty of 0.5° causes a standard gain uncertainty of 1.6 dB. Thus the standard gain uncertainty using the Δ probe is greater than 1.8 dB.

These probes all have their patterns down by about 20 dB from their peak at wide angles, hence the standard uncertainty in the amplitude of the AUT’s pattern at wide angles is about 0.3 dB due to uncertainties in the relative patterns of the probes.

### 5.2 Near-field Measurement Uncertainties

In addition to the uncertainties in the probe parameters, there are uncertainties in the calculated far field due to uncertainties in the near-field measurements.

A number of these uncertainties cannot be estimated beforehand. They are: (1) the multiple reflection effects because there exists no theoretical method to determine the magnitude of the multiple reflections between probe and AUT; (2) the impedance uncertainties (which affect only the gain) because the reflection coefficients and their uncertainties are unknown until measured; (3) uncertainties due to leakage and crosstalk until they are actually measured; and (4) uncertainties due to room scattering. Room scattering has not played an important role at the NIST facility in the past because its magnitude is small but it could be a limiting factor in measuring low sidelobes. For the low-sidelobe measurement, NIST developed a test to estimate the effect of room scattering as was described earlier. The implications of the results of this test for the measurement accuracy will be discussed later.

The effect of multiple reflection can be estimated by taking measurements at several *Z*-distances, averaging the results, and subtracting the average from the individual measurements. The results of these tests were discussed in a previous section. In short, the multiple reflection uncertainties were greater for the difference probe than for the other two probes that were used in the measurement and were a function of far-field angle.

Impedance uncertainties were small and contributed at most 0.05 dB to the uncertainty in the gain of the AUT.

Leakage and crosstalk were estimated by making one-dimensional near-field measurements first with a termination on the transmitting side with the probe operating normally and then with a termination on the receiving side with the AUT operating normally. The leakage and crosstalk were 75 dB below the near-field peak and cause uncertainties in the far-field pattern as summarized in Table 6.

As indicated in Sec. 4, the peak room scattering is −70 dB, and the RMS room scattering is −89 dB. From this, we obtain the uncertainties in Table 7.

In principle, data point spacing can be chosen so that aliasing is arbitrarily small. However, noise and/or rapidly varying systematic errors (for example, multiple reflections) set the practical lower limit. If the data spacings in *X* and *Y* are *δ_x_* and *δ_y_* respectively, then the aliased Fourier transform of the data *F*_e_(*K*) in terms of the true FFT is [7]

$$F_{e}\left(K\right)=\sum_{m,\;n\;=\;-\infty }^{\infty }F\left(k_{x}+\frac{2m\;\pi }{\delta_{x}},k_{y}+\frac{2n\;\pi }{\delta_{y}}\right).$$ (12)

Aliasing is contributed by terms for which *m* ≠ 0, *n* ≠ 0. The terms *m* = ±1, *n = ±*1 are usually the only terms which contribute significantly to this error. The magnitude of the aliasing uncertainty can be estimated using the test space procedure. In this test a centerline near-field scan is performed using very small spacing between data points. The FFT is then performed on the complete set of data, then on every other data point, then every third point, and so forth. Using these centerline tests we are able to determine a data point spacing such that the aliasing error is small in comparison to other errors for all three probes. The aliasing uncertainty is about the same for all of the probes.

**Fig. 30:**
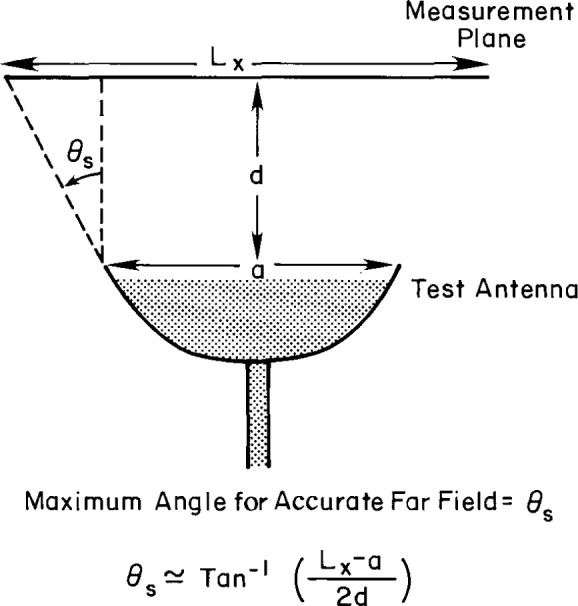
Schematic relationship between the scan length and the maximum angle to which far-field patterns can be accurately computed.

Area truncation has two effects. First, the far-field pattern results obtained by Fourier transforming the PNF data are valid only within the angular region defined by the geometry of the antenna and the scan area, as shown in Fig. 30. The second effect produces uncertainties in the far-field pattern within the region of validity. Yaghjian [6] showed that we can obtain an upperbound uncertainty from a knowledge of the measured data on the boundary of the scan area. Denoting the plane-polar coordinates of the boundary by (*p′, ϕ*_p_), the normalized data on the boundary *by B*(*p′, ϕ_p_*), the spherical coordinates in the far field by (*θ, ϕ*), the magnitude of the electric field by |*E*(*r*)|_r_*_→∞_*, and the maximum acute angle between the plane of the scan area and a line connecting the edges of the antenna aperture and the scan area by *γ*_max_, we express the fractional uncertainty in the far field as

$$\left|\frac{\Delta D\left(\theta ,\phi \right)}{D\left(\theta ,\phi \right)}\right|\leq \frac{\left|\int_{0}^{2\pi }B\left(p^{\prime },\phi_{p}\right)e^{-ikp^{\prime }\sin\theta \cos\left(\phi -\phi \right)}p^{\prime }d\phi_{p}\right|}{2\pi r{\left|E\left(r\right)\right|}_{r\to \infty }\cos\gamma_{\max}}.$$ (13)

In this test an FFT is done on the full set of data, then all the data are set to zero except the data at the edges and the FFT recomputed. The far fields from these two data sets can be compared and used to determine the uncertainty due to the truncation of the scan plane. We find that the standard truncation uncertainty for the peak of the far field is 0.06 dB. At 40 dB below the far-field peak the standard truncation uncertainty is about 0.3 dB and at 60 dB below the far-field peak the standard uncertainty is 0.9 dB.

The remaining uncertainties, which are due to uncertainties in position, amplitude, phase, and alignment, were shown by Yaghjian [6] and Newell [7] to have a term in common: they all depend on the ratio *g*(*K*) of the peak uncorrected far-field amplitude *D*(*K*_0_) to the far-field amplitude *D*(*K*) in the direction *K.* Because the difference probe has a null in the direction of the AUT’s main beam it leads to an uncorrected far field which has a peak value which is 25 dB to 30 dB below the peak of both the sum probe and the open-end waveguide. As a result these uncertainties will be 25 dB to 30 dB below the corresponding uncertainties for the other two probes.

Position uncertainties (and some other uncertainties) often concentrate their effects in certain directions because of periodicities in the measurement (for example, the structural supports of the scanner). If there is no periodicity then these uncertainties will add to the noise of the far field. Otherwise, the position uncertainties are concentrated in the directions sin (*γ*/*τ*_i_) relative to the direction of the main beam, where *τ*_i_ is the corresponding periodicity. For antennas in which the main beam is steered off axis and for direction angles near the main beam, (*θ−θ*) < *γ*/(10*L*) (*L* is the maximum antenna dimension and *θ*_b_, is the angle between the direction of the *Z*-axis and the direction of the main beam) the uncertainty in the far field is [7]:

$${\left|\frac{\Delta D\left(K\right)}{D\left(K\right)}\right|}_{\text{dB}}\leq \frac{344}{\sqrt{\eta }}{\left(\frac{\Delta \left(K\right)}{\lambda }\right)}^{2}\sin^{2}\theta_{b}\;g\left(K\right)$$ (14)

for *X* and *Y* uncertainties, and

$${\left|\frac{\Delta D\left(K\right)}{D\left(K\right)}\right|}_{\text{dB}}\leq \frac{43}{\sqrt{\eta }}{\left(\frac{\delta_{z}\left(K\right)}{\lambda }\right)}^{2}\cos^{2}\theta_{b}\;g\left(K\right)$$ (15)

for the Z uncertainties. For directions where λ/(10*L*) < (*θ*−*θ*_b_) < π/2 the uncertainty is

$${\left|\frac{\Delta D\left(K\right)}{D\left(K\right)}\right|}_{\text{dB}}\leq 13.5\left(\frac{\Delta \left(K\right)}{\lambda }\right)\sin\theta_{b}\;g\left(K\right)$$ (16)

for the *X* and *Y* uncertainties, and

$${\left|\frac{\Delta D\left(K\right)}{D\left(K\right)}\right|}_{\text{dB}}\leq 13.5\left(\frac{\delta_{z}\left(K\right)}{\lambda }\right)\cos\theta_{b}\;g\left(K\right)$$ (17)

for the *Z* uncertainties. *η* is the aperture efficiency, Δ(*K*) is the FFT of the *X* and *Y*-position errors, and *δ*_Z_ (*K*) is the FFT of the Z uncertainties. Table 8 shows the period and magnitude of the position errors that are observed for the NIST near-field scanner.

The period of 9.1 m in Table 8 corresponds approximately to twice the scanner’s height, and 40.5 cm corresponds to the distance between supports in the *X*-direction. For *L* = 6 m, the largest dimension of the ULSA, η = 0.5, *θ*_b_ = 12.7° (the location of the ULSA main beam), we obtain the uncertainties found in Table 9 for the sum and open-end waveguide probes.

For the difference probe, a −40 dB sidelobe would have *g*(*K*) = 3.2 instead of 100 as is the case for the other two probes and a −60 dB sidelobe corresponds *to g*(*K*) = 32 instead of 1000. This is a consequence of the 30 dB null which the difference probe has in the direction of the AUT’s main beam and which reduces the peak value of *D* (*K*_0_) by 30 dB. The resulting uncertainties for the difference probe are found in Table 10.

Because of the difference probe’s properties the position uncertainties for a −40 dB sidelobe and a −60 dB sidelobe are greatly reduced in comparison to both the sum probe and the open-end waveguide probe.

Amplitude and phase instrumentation uncertainties arise from receiver nonlinearity, flexing of cables and rotary joints, source and receiver drift, and temperature drift. The drift amounts to about 0.5°/h. However, we can partially correct for drift by the use of tie scans. There is also a 2° to 3° phase uncertainty associated with the flexing of cables to the probe and with the rotary joint. The periods for the cable flexing are associated with the *X* and *Y* dimensions of the scanner, about 4.5 m in each direction. We can partially correct for receiver nonlinearity by using a calibration of the receiver against a calibrated rotary vane attenuator. Using this calibration we can make a first-order correction for the nonlinearity of the receiver. The first-order correction coefficient for the frequency band of the ULSA is 0.01 ± 0.005. Thus 0.005 is the peak residual uncorrected nonlinearity. The periods associated with this uncertainty depend on the variation of the near-field amplitude and phase as functions of *X* and *Y.* The near-field amplitude variation depends partly on the properties of the probe. The expected periods for the sum and open-end waveguide probes are 8 m in *X* and 2 m in *Y.* The expected periods for the difference probe are 8 m in *X* and 1 m in *Y.* The upperbound amplitude uncertainty due to nonlinearity is [7]

$${\left|\frac{\Delta D\left(K\right)}{D\left(K\right)}\right|}_{\text{dB}}\leq 6U\left(K\right)g\left(K\right),$$ (18)

where *U*(*K*) is the FFT of the residual receiver non-linearity uncertainty. When λ/(10*L*) ≤ (*θ*−*θ*_b_) ≤ − π/2 the uncertainty in the phase nonlinearity causes a far-field uncertainty which is [7]

$${\left|\frac{\Delta D\left(K\right)}{D\left(K\right)}\right|}_{\text{dB}}\leq 13.5\frac{V\left(K\right)}{360}g\left(K\right),$$ (19)

where *V*(*K*) is the FFT of the residual phase uncertainties (including the uncertainty due to cable flexing). The resulting far-field uncertainties due to residual uncorrected receiver nonlinearity in the phase and amplitude are shown in Table 11. The uncertainties of Table 11 for the −20 dB and −40 dB sidelobes are less for the Δ probe but not much less because the largest uncertainties lie in the direction of this probe’s null.

In addition to the above mentioned phase and amplitude uncertainties, there is also round-off which will contribute to the noise. The near-field amplitude is measured on a 0 to 100 linear scale; the maximum round-off for the amplitude is 0.05 on this scale. The phase is measured in degrees and has a maximum round-off of 0.05°. The signal-to-noise ratio due to the amplitude round-off is given by Newell [7] as

$${\left(\frac{S}{N}\right)}_{a}=\frac{\sqrt{N_{e}/2}}{\left(N/N_{e}\right)3\sigma_{a}},$$ (20)

and due to the phase round-off as

$${\left(\frac{S}{N}\right)}_{\text{ψ}}=\frac{\sqrt{N_{e}/2}}{3\sigma_{\text{ψ}}},$$ (21)

where *N*_e_ is the number of measurement points within the effective area, *N* is the total number of measurement points, and the *σ*’s are the standard deviation of the round-off distributions. Since we used a spacing of 3.81 cm in both *X* and *Y* and a measurement area of about 10.4 m in × by 3.8 m in *Y*, then *N* ≈ 27 500 measurement points. Assuming a 50% efficiency for the antenna and using the dimensions of the ULSA we find *N*_e_ ≈ 2500. With maximum round-off of 0.05 for the amplitude and 0.05° for the phase then *σ*_a_ = 0.0003 relative to the peak and *σ*_ψ_ = 0.0005 (in radians) and we find (*S*/*N*)_a_ = 82 dB and (*S*/*N*)_ψ_ + = 87 dB. The total signal-to-noise ratio is therefore expected to be about 81 dB.

A summary of the approximate uncertainties as derived from the above tests and analyses is given in Table 12. Assuming these uncertainties are independent of each other the total standard uncertainty is the root sum square (RSS) of these errors. This implies that the total standard uncertainty at −55 dB for the waveguide probe is (+3.2 dB, −5.1 dB) and that for the difference probe is (+2.2 dB, −3.0 dB). Uncertainties will exceed this in some directions where periodicities are larger.

Our analysis for the NIST near-field facility leads us to conclude that it should be possible to accurately measure (within a few decibels) sidelobes down to about 55 dB below the AUT’s peak far field.

### 5.3 Comparison of Near-Field and Far-Field Results

**Fig. 31:**
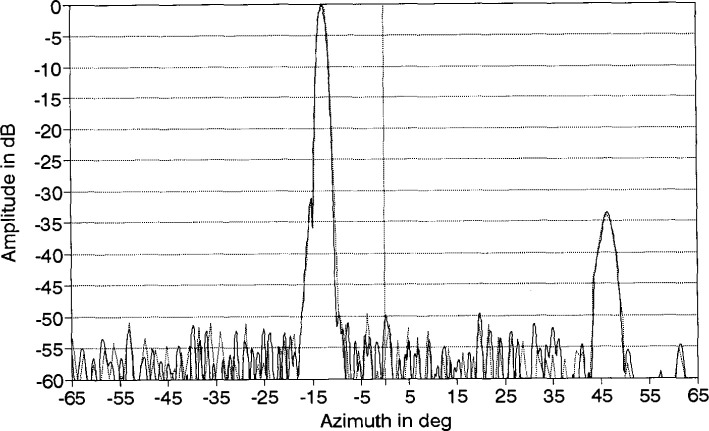
Comparison of far-field patterns determined using the PNF range (solid) and the far-field range (dotted line).

We can now compare the far-field patterns obtained from the NIST PNF range to those obtained by others on a far-field range. When we overlay the results of Fig. 27 to those in Fig. 3 we found that there is an offset between the two patterns of 0.5°. Accounting for this offset and overplotting the two results (Fig. 31) we see the two patterns agree quite well. At −55 dB the difference between the two patterns is generally less than 5 dB, which is within the NIST measurement uncertainty.

## 6. Future Research

If we could ignore the multiple reflections caused by the difference probe, this probe would clearly be the best probe to use for these low-sidelobe PNF measurements (but not for the gain or main beam). However, the effects of multiple reflections are substantially worse for the Δ probe than for the other two probes so the Δ probe proves to be only slightly better (about 2 dB) in the level to which it is able to distinguish the low sidelobes of the AUT.

Why a probe or any antenna has particular reflection properties is poorly understood. There is only a rough approximation for correcting for this effect. The argument is usually made that if data are taken over enough scan planes and transformed to the far field while accounting for the different separation distances, and data from the various planes averaged, then the multiple reflection effects tend to cancel. There is no test for determining how many different scan planes are adequate for this procedure. In calculating the far field from near-field data, the terms involving multiple reflections between the AUT and the probe are ignored.

It would be desirable to approach the problem of multiple reflections from both the experimental and theoretical views. From the theoretical view the possibility of including the first-order multiple reflection term could be explored. According to Kerns [12] the full solution to the transmission equation is

$$\frac{b_{0}^{\prime }}{a_{0}}={{\hat{S}}^{\prime }}_{02}\;{\hat{T}}_{21}\left(1-{\hat{S}}_{11}{\hat{R}}^{\prime }\right){\hat{S}}_{10},$$ (22)

where ${\hat{R}}^{\prime }$ describes the receiving probe as a passive scatterer in the transmitting coordinating system, and ${\hat{S}}_{11}$ describes the scattering properties of the transmitting antenna. In practice for near-field measurements we assume ${\hat{S}}_{11}{\hat{R}}^{\prime }\sim 0$. Since multiple reflections are obviously present, often it would be desirable to know what can be done to solve equation (6-1) when ${\hat{S}}_{11}\;{\hat{R}}^{\prime }\neq 0$.

From an experimental view point it would be desirable to know which kinds of probes and antennas produce larger multiple reflections. This might be determined by doing *Z*-multiple-reflection tests (as described in Sec. 3.3) for different combinations of AUT and probe.

## 7. Summary

The near-field measurement technique can be used to measure sidelobes of very low-sidelobe antenna arrays. Near 3 GHz the NIST measurement facility can measure sidelobes to about 55 dB to 60 dB below the AUT’s peak far field. The main limitations to accurately determining sidelobes below 60 dB are multiple reflection effects between the AUT and the probe.

The Δ probe can be useful in reducing some uncertainties, which depend on the quantity *g*(*K*). However, because higher multiple-reflection effects are associated with it, it is only marginally better than an open-end waveguide in measuring low sidelobes. If multiple reflections could be substantially reduced, the difference probe would be an extremely useful probe to measure sidelobes below 65 dB.

The Δ probe cannot be used to accurately determine the main beam region of the AUT far-field pattern. This is because in the region of the Δ probe null, which corresponds to the AUT main beam, small uncertainties in steering cause large uncertainties (1.5 dB or more) in the probe pattern amplitude which in turn causes large uncertainties in the probe correction for the AUT main beam.

## Figures and Tables

**Table 1:** Summary of two-dimensional near-field measurements

AUT	Frequency (GHz)	Open-end Waveguide	Δ	Σ
ULSA	2.9		*M, Z* = 65 cm	*M, Z* = 65 cm
ULSA	3.0	*M, Z* = 65 cm		
ULSA	3.1	*M, Z* = 65 cm	*M,Z* = 65 cm	*M, Z* = 65 cm
ULSA	3.1	*C, Z* = 65 cm		
AWACS	F2	*M, Z* = 35 cm	*M, Z* = 35 cm	
AWACS	F2	*C, Z* = 35 cm		
AWACS	F2	*M,Z* = 37.5 cm	*M,Z* = 37.5 cm	

Δ = measurement with difference probe.

Σ = measurement with sum probe.

*Z* = the probe-antenna separation distance.

*M* = measurement of main component.

*C* = measurement of cross component.

**Table 2:** Predicted and measured errors due to simulated *Z*-position errors

Period	Amplitude	Probe	Measured location (*k_x_*/*k*)	Predicted Δ*D* (dB)	Measured Δ*D* (dB)
2.5 λ	0.13 mm	Σ	0.23	9.5	9.5
			−0.57	7.9	8.6
1.67 λ	0.25 mm	Σ	0.43	9.6	11.9
			−0.77	7.0	8.6
1.11 λ	0.38 mm	Σ	0.73	7.7	8.8
2.5 λ	0.13 mm	Δ	0.23	0.3	0.2
			−0.57	0.25	0.3
1.67 λ	0.25 mm	Δ	0.43	0.3	0.2
			−0.77	0.2	0.3
1.11 λ	0.38 mm	Δ	0.73	0.2	0.3

**Table 3:** Predicted and measured signal-to-noise ratios for AWACS

Probe type	Array	Signal/Noise ratio Measured^a^ (dB)	Spectral ratio Measured^b^ (dB)
Open-end W/G	AWACS	82.2	74.2
Delta	AWACS	94.0	89.7

a Excluding correlated evanescent lobe ridges.

b All evanescent space.

**Table 4:** Uncertainty sources in planar near-field measurements

1.	Probe relative pattern
2.	Probe polarization ratio
3.	Probe gain measurement
4.	Probe alignment
5.	Normalization constant
6.	Impedance mismatch factor
7.	Antenna alignment
8.	Data point spacing (aliasing)
9.	Measurement area truncation
10.	Probe X–Y position uncertainties
11.	Probe *Z*-position uncertainties
12.	Multiple reflections (probe/AUT)
13.	Receiver amplitude nonlinearity
14.	System phase uncertainty
	Receiver phase nonlinearity
	Flexing cables and rotary joints
	Temperature effects
15.	Receiver dynamic region
16.	Room scattering
17.	Leakage and crosstalk
18.	Round-off in amplitude / phase

**Table 5:** Typical probe uncertainties

Source of uncertainty	Standard uncertainty (in dB)
Probe gain	0.06
Relative probe pattern amplitude	
at −5 dB	0.09
at −15 dB	0.2
at −30 dB	0.9

**Table 6:** Far-field pattern uncertainties due to leakage

Pattern amplitude (in dB)	Standard uncertainty (in dB)
0.	0.0004
−15.	0.003
−30.	0.02
−45.	0.1
−60.	0.2
−75.	2.4

**Table 7:** Uncertainties due to room scattering

Pattern amplitude (in dB)	Standard uncertainty (in dB)
0.	0.0003
−15.	0.002
−30.	0.01
−45.	0.06
−60.	0.3
−75.	1.9

**Table 8:** Mechanical periods for the NIST scanner

Type of uncertainty	As a function of	Periods	Magnitude (in cm)
*Z*	*X*	none	0.04
	*Y*	9.1 m	0.06
*Y*	*X*	40.5 cm	0.01
		91.0 cm	0.01
*X*	*Y*	9.1 m	0.06

**Table 9:** Far-field uncertainties resulting from near-field position uncertainties using sum and open-end waveguide probes

Type	Function variable	Direction angle from main beam	Sidelobe level (in dB)	Standard uncertainty (in dB)
*Z*	*X*			noise
	*Y*	±0.6	0.0	0.05
*Y*	*X*	±6.0	−40.0	0.2
		±14.0	−60.0	1.7
*X*	*Y*	±0.6	0.0	0.01

**Table 10:** Far-field uncertainties resulting from near-field position uncertainties using the difference probe

Type	Function variable	Direction angle from main beam	Sidelobe level (in dB)	Standard uncertainty (in dB)
*Z*	*X*			noise
	*Y*	±0.6	0.0	0.08
*Y*	*X*	±6.0	−40.0	0.01
		±14.0	−60.0	0.09
*X*	*Y*	±0.6	0.0	0.02

**Table 11:** Far-field uncertainties due to residual amplitude and phase uncertainties

Type	As a function of	Direction from main beam	Sidelobe level (in dB)	Standard uncertainty (in dB)
Amplitude	*X*	±0.7	0.	0.03
	*Y*	±1.3	−20.	0.3
		±3.0	−40.	3.0
Phase	*X*	±1.3	−20.	1.0
	*Y*	±1.3	−20.	1.0

**Table 12:** Far-field uncertainties using the NIST planar scanner

	Standard uncertainties in dB for a −55 dB sidelobe	
Uncertainty source	Waveguide probe	Difference probe
Truncation	+1.4	±0.5
	−1.6	
Aliasing	±0.5	±0.5
Multiple reflections	+1.4	+2.2
	−1.6	−2.9
Room scattering	±0.2	±0.1
Position	+2.2	±0.1
	−2.9	
Amplitude	+1.4	±0.1
	−1.6	
Phase uncertainty	+1.4	±0.1
	−1.6	
Random noise	±0.4	±0.3
RSS uncertainty	+3.2	+2.2
	−5.1	−3.0
